# Mechanisms of Action of Instillation and Dwell Negative Pressure Wound Therapy with Case Reports of Clinical Applications

**DOI:** 10.7759/cureus.3377

**Published:** 2018-09-27

**Authors:** Mario A Aycart, Danielle J Eble, Kimberly M Ross, Dennis P Orgill

**Affiliations:** 1 Plastic Surgery, Brigham and Women's Hospital, Boston, USA; 2 Plastic Surgery, Boston University School of Medicine, Boston, USA; 3 Plastic Surgery, Brigham & Women’s Hospital, Boston, USA

**Keywords:** negative pressure wound therapy, wounds, mechanism of action, wound healing, wound complications, instillation vac

## Abstract

Negative pressure wound therapy (NPWT) has revolutionized the care of complex wounds since 1997. The addition of instillation (NPWTi-d) adds several potential benefits and challenges to clinicians dealing with complex wounds in a hospital setting.

We surveyed the literature regarding the mechanism of action of these devices and reviewed our clinical experience to date.

Potential mechanisms of action include the removal of microorganisms from the wound surface, dilution of cytotoxic molecules, upregulation of angiogenesis pathways, and maintenance of a moist wound environment. As we have extended our use of these devices to more complex wounds, we have taken advantage of and observed potential mechanisms of action, including facilitated removal of microorganisms, dilution of inflammatory and cytotoxic macromolecules, additional wound hydration, and enhanced angiogenesis through an intermittent application of NPWT. We have also observed complications, including bleeding, loss of a seal along the wound, and pain.

NPWTi-d provides additional options for clinicians caring for complex wounds with favorable responses in wounds with significant contamination and those with poor inherent vascularity. Further studies to clarify the mechanisms of action, better define the wound types that would benefit, and techniques to manage complications using this device should further advance this field.

## Introduction

Negative pressure wound therapy (NPWT) was introduced in 1997 and rapidly became the treatment of choice for many complex wounds [1-2]. NPWT devices consist of a porous interface material that efficiently transmits pressure, a semi-occlusive covering, and tubing that connects to a suction source [2]. Clinicians were sufficiently impressed with the dramatic response seen in wounds treated with NPWT that it became difficult to maintain clinical equipoise and enroll patients in prospective randomized controlled trials (RCT) as clinicians did not want their patients to be randomized to the control. As a result, there are few robust, high-level clinical trials attesting to the efficacy of this device and now, over 20 years since the seminal work, a robust RCT is underway [3-6].

We have previously published numerous pre-clinical studies to better describe the mechanisms of action of NPWT devices, focusing our efforts on devices with porous polyurethane foam [7-11]. We proposed four basic mechanisms of action: 1) macrodeformation or wound shrinkage, 2) microdeformation or micromechanical cellular changes at the wound-interface surface, 3) removal of fluids, and 4) maintenance of a moist wound environment [7]. We showed that the kinetics of granulation tissue formation are related to the size of the pores in the foam, the level of suction applied, and the stiffness of the wound [8-9]. We also found that granulation tissue formation is affected by the time and frequency of application [10-11].

Shortly after the advent of NPWT, reports emerged of gravity instillation of fluids that were allowed to be instilled into the foam and allowed to dwell (NPWTi-d) as an adjunct to therapy [12]. Clinicians started using intermittent instillation of fluids to treat a variety of acute and chronic wounds in small case series using various antiseptic and antibacterial solutions [13]. At that time, many felt the addition of antimicrobial agents in these solutions was a critical component of the success observed. In France, where these solutions are not permitted for use, Teot et al. showed promising results with normal saline [14]. Kim et al. were able to suggest comparable outcomes in a prospective, randomized, comparative effectiveness study using 0.9% normal saline versus 0.1% polyhexanide, plus 0.1% betaine solution [15]. Based on these experiences and published guidelines, we currently use 0.9% normal saline in our practice and will focus this paper on the use of normal saline instillation with the NPWTi-d system. We describe our proposed mechanisms of action and highlight our clinical experience using the NPWTi-d system.

## Case presentation

We reviewed the published literature on both the mechanism of action and clinical usage of NPWTi-d. We used the search term "negative pressure wound therapy instillation" in PubMed without date, language, or type of publication restrictions from date of inception to August 10, 2018. The device used in our institution is the V.A.C. Ulta™ with V.A.C. VERAFLO™ Instillation Therapy (Kinetic Concepts, Acelity, San Antonio, TX). We typically use about one-third of the wound volume to instill saline and observe the color of the foam uniformly turn to a darker black. We initially base this on the dimensions of the wound but adjust the volume based on how much of the foam becomes hydrated. We allow this to dwell for 10 minutes and then remove at 125 mm Hg of suction to the collection canister unless otherwise specified. This is repeated every two hours, similar to the published guidelines. We also reviewed our clinical experience to date using NPWTi-d and include cases that demonstrate our proposed mechanisms of action and highlight the management of our observed complications.

Mechanism of action NPWTi-d

**Figure 1:**
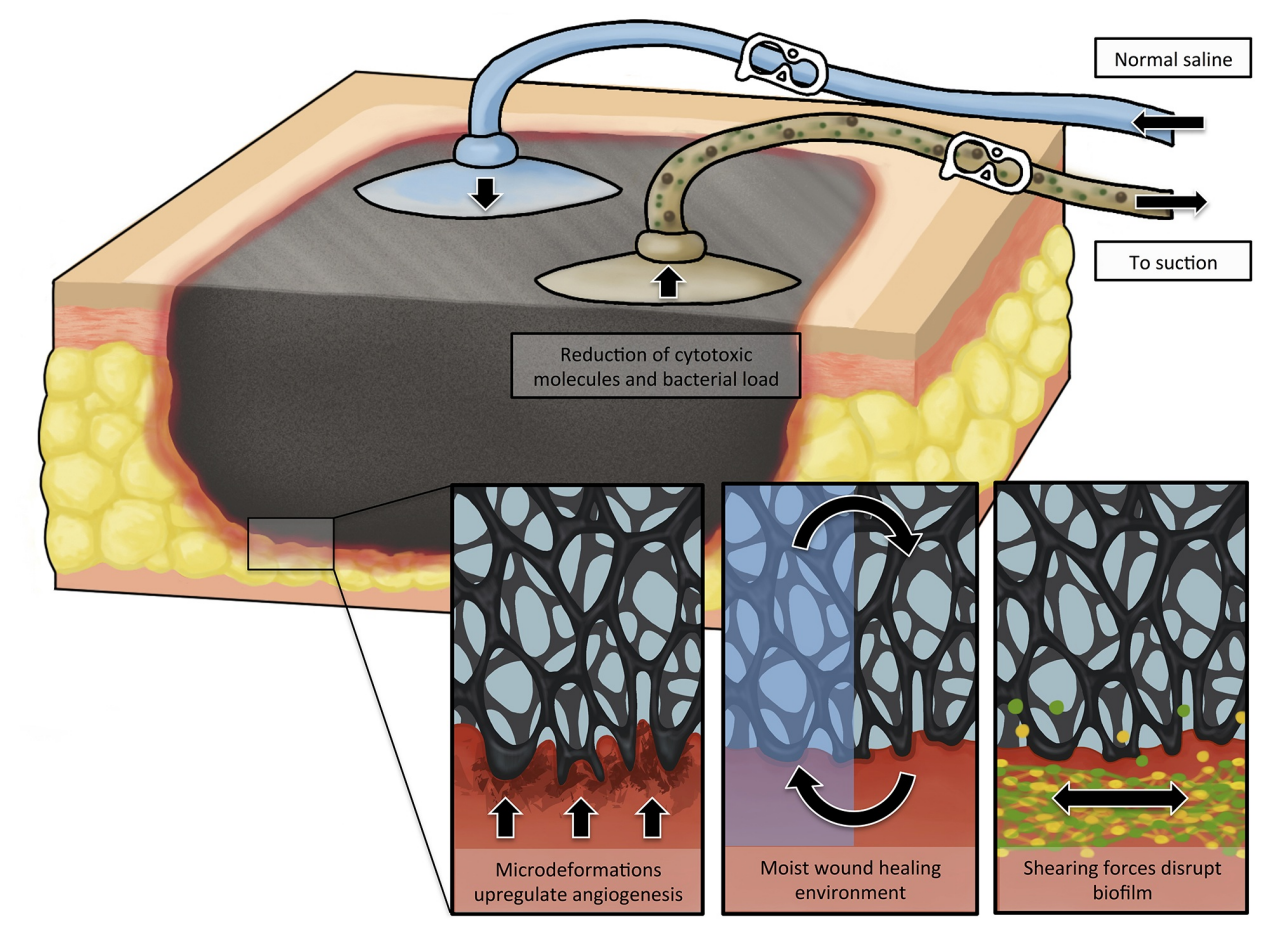
Schematic representation of mechanisms of action of NPWTi-d. In addition to the standard NPWT mechanisms, NPWTi-d adds other potential mechanisms, including facilitated removal of microorganisms, dilution of inflammatory and cytotoxic macromolecules, additional wound hydration, and enhanced angiogenesis through intermittent application of NPWT. Note that in some configurations of NPWTi-d, the instillation and suction functions are integrated into one port. NPWT: negative pressure wound therapy; NPWTi-d: negative pressure wound therapy with instillation and dwell time

In addition to the four principle mechanisms of action described for traditional NPWT, the literature and our clinical experience suggest four additional mechanisms for NPWTi-d: 1) facilitated removal of microorganisms, 2) dilution of inflammatory and cytotoxic macromolecules, 3) additional wound hydration, and 4) enhanced angiogenesis through intermittent application of NPWT (Figure 1) [7].

Facilitated removal of microorganisms

NPWT allows for the removal of bacteria and wound exudate, though its influence on the reduction of the bacterial bioburden remains inconsistent [7]. Wound irrigation is believed by many to be a basic principle in the management of contaminated wounds. On a macroscopic level, we can observe the cleansing of wound exudate and debris from grossly contaminated wounds. As a natural extension of irrigation, instillation allows for the even distribution of a solution over the wound surface that can reach undermined and tunneled regions of a wound [16]. Furthermore, NPWTi-d has been shown to effectively remove debris while significantly limiting cross-contamination and bacterial aerosolization when compared to low-pressure lavage systems in a pre-clinical study [17]. The saline allows microorganisms, both at the wound surface and in the foam, to be removed. During this process, the microdeformation of the wound occurs when applying and removing suction causing small movements of the pores in relation to the wound surface which may break up biofilm, wound exudate, and necrotic material. The open cell reticulated foam with through holes appears as a large honeycomb pore array. This is manufactured as part of the V.A.C. VERAFLO Cleanse Choice™ dressing (Acelity, San Antonio, TX) which appears to be particularly effective for removal of necrotic material. In addition, its large pores allow areas within the honeycomb (through pores) to develop robust granulation tissue [14].

Case 1: Infected Achilles Wound 

**Figure 2:**
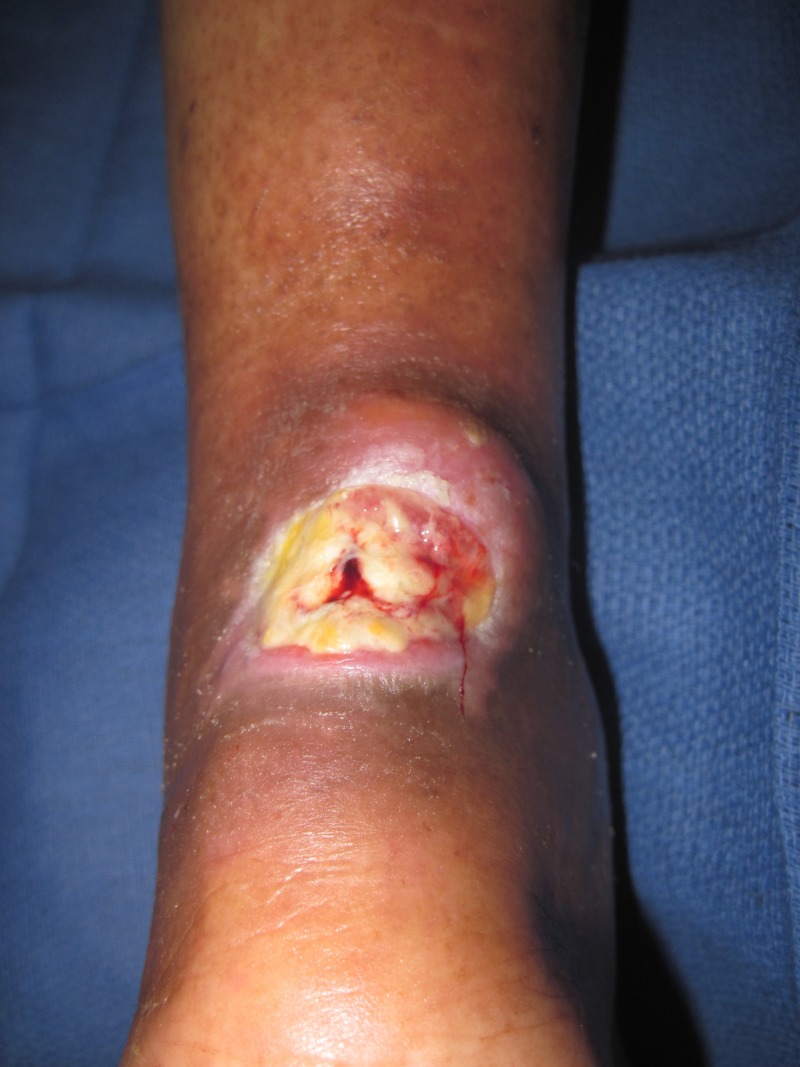
Chronic Achilles wound presents with fever and chills. Note the lack of inflammation over the posterior lower leg.

**Figure 3:**
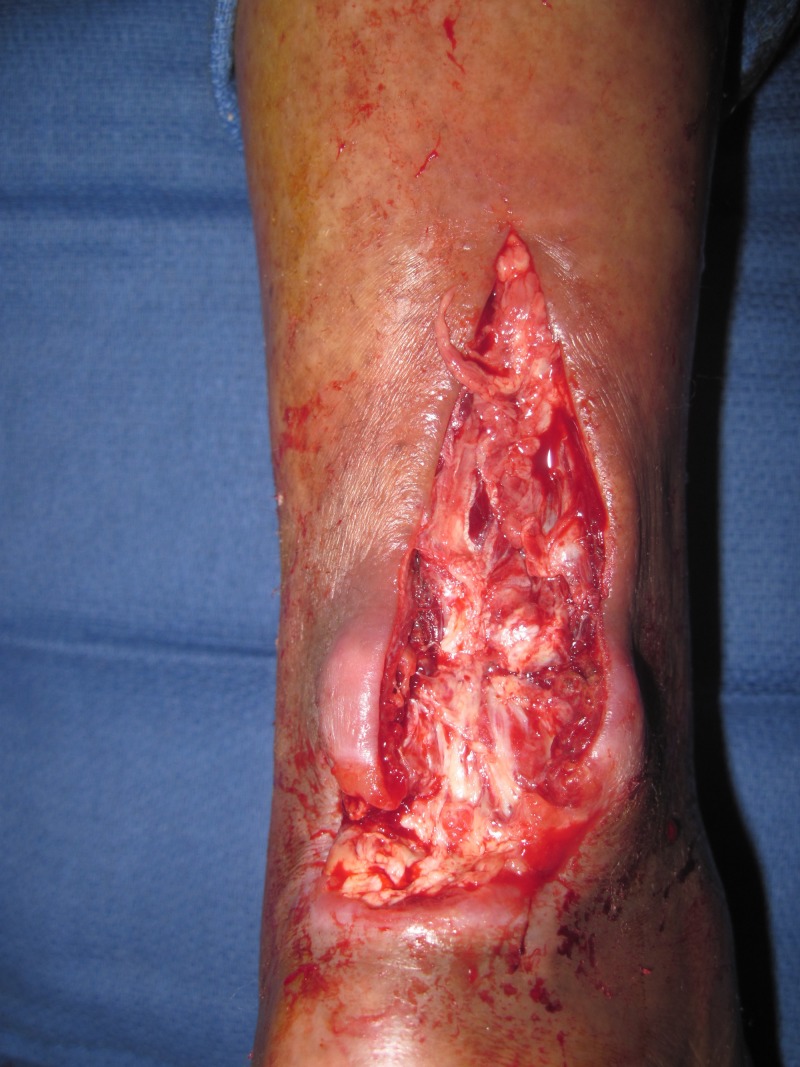
Following incision and drainage. Note the large area of destruction of portions of the Achilles tendon.

**Figure 4:**
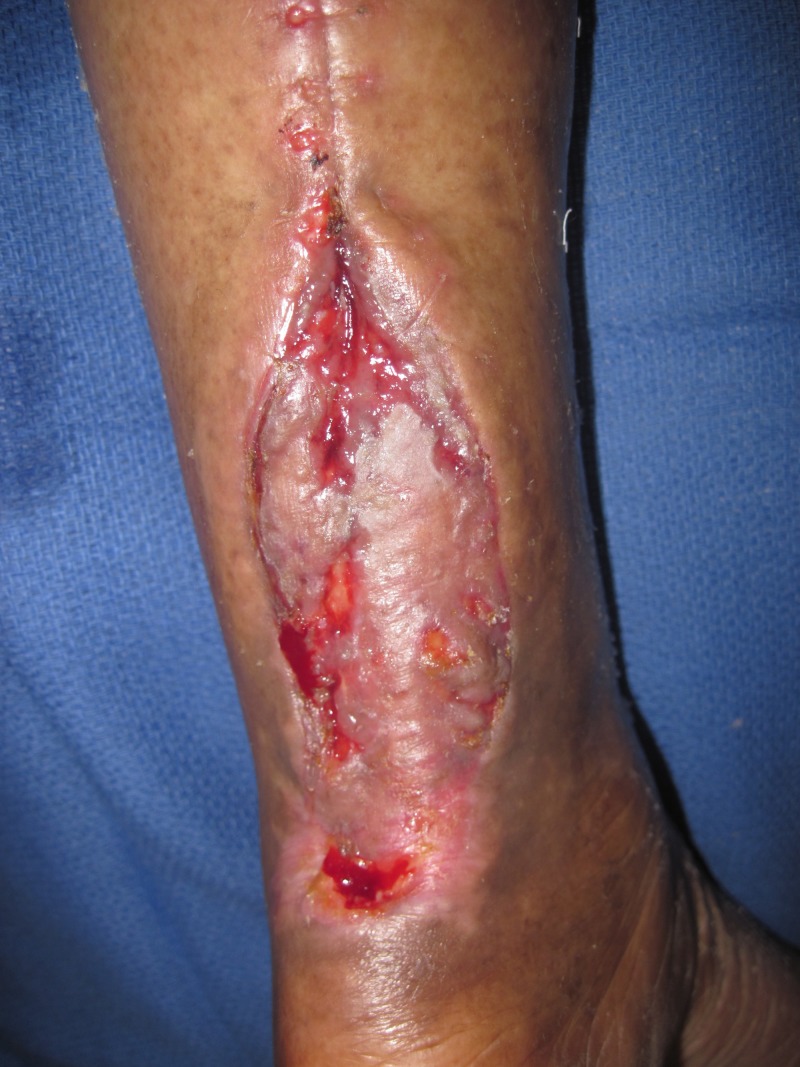
Treated with NPWTi-d and skin graft. Note a good skin graft has taken over much of the tendon. NPWTi-d: negative pressure wound therapy with instillation and dwell time

**Figure 5:**
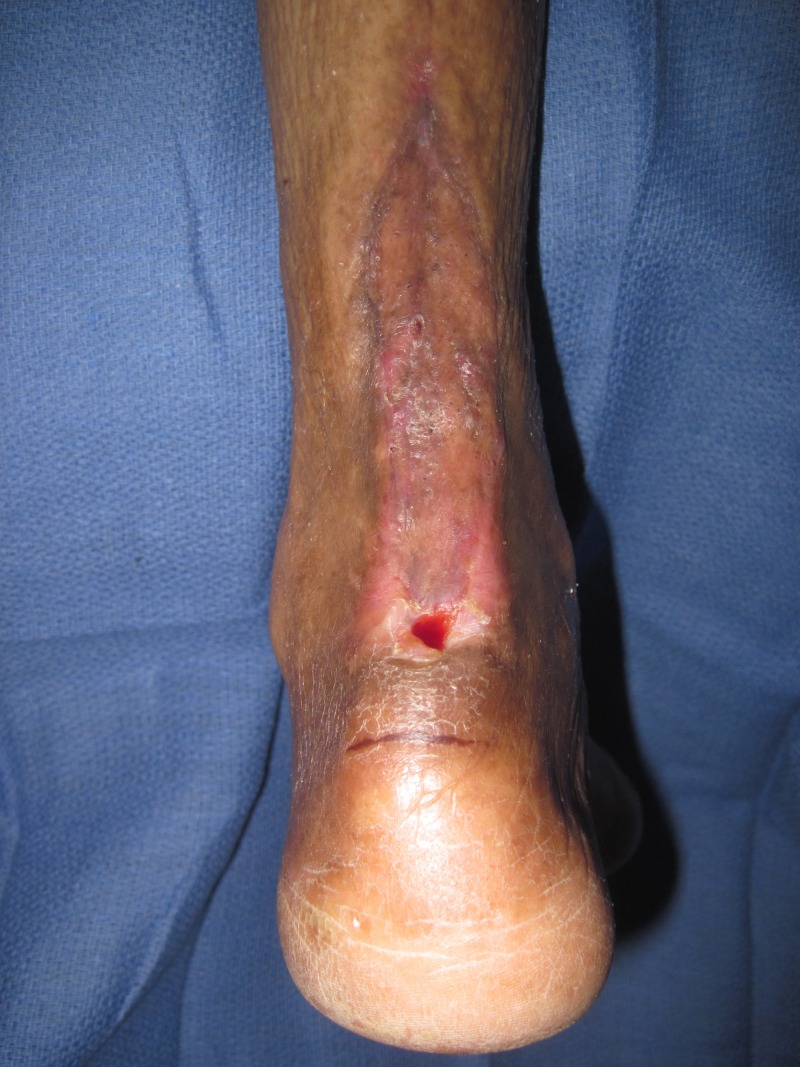
Two months after treatment.

A 62-year-old female presented with a left Achilles wound in the context of severe peripheral vascular disease status-post revascularization of bilateral lower extremities. Comorbid conditions included obesity, insulin-dependent diabetes, and heart failure. She presented with severe pain, redness, and swelling of her lower extremity and a 4 x 4 x 0.5 cm wound on her left Achilles, probing along the tendon for over 15 cm. She was taken to the operating room (OR) immediately, and under local anesthesia, a large abscess extending to the mid-portion of the gastrocnemius muscles was drained. She returned to the OR two additional times where the V.A.C. VERAFLO™ was placed and the wound subsequently surgically closed. The foam was instilled with normal saline, 20 cc every two hours, which was allowed to dwell for 10 minutes. This case demonstrates how the bacterial load was reduced and the wound optimized, allowing for wound closure (Figures 2-5).

Dilution of inflammatory and cytotoxic macromolecules

Wound healing typically follows a highly regulated, predictable pattern that is usually characterized by overlapping phases. These include hemostasis, inflammation, proliferation, and remodeling. Chronic, non-healing wounds do not follow this pattern of normal healing, and prior studies have elucidated these mechanisms, including elevated levels of inflammatory cytokines and proteases with low levels of growth factors [18-19]. Specifically, these wounds show elevated levels of metalloproteinases (MMP) which have been suggested to play a key role in poor wound healing [19]. One of the key proposed mechanisms of NPWT is the removal of fluid and, in the case of NPWTi-d, dilution of cytotoxic and inflammatory molecules. Studies have shown wounds treated with NPWT to have lower levels of MMPs and increased expression of leukocyte chemoattractants [20].

Case 2: Upper Extremity Necrotizing Soft Tissue Infection

**Figure 6:**
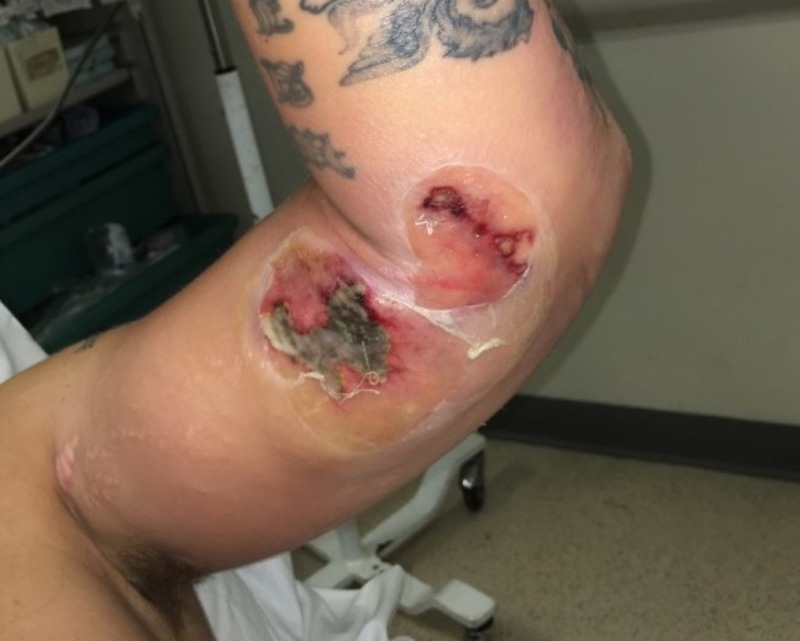
Necrotizing fasciitis of the elbow. Note blistering of the skin.

**Figure 7:**
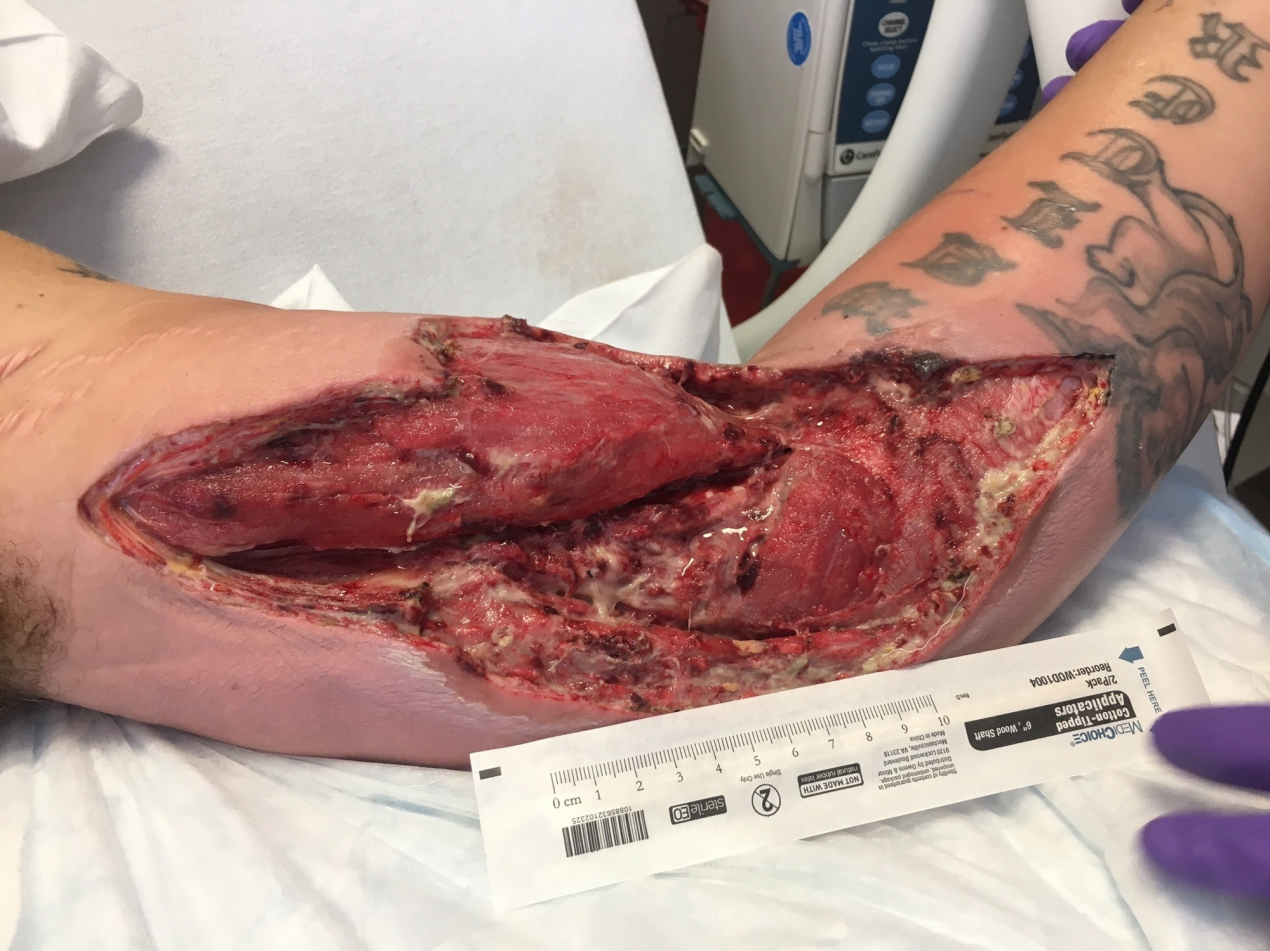
Following radical debridement. Note the substantial swelling of the extremity with exposure of the brachial vessels.

**Figure 8:**
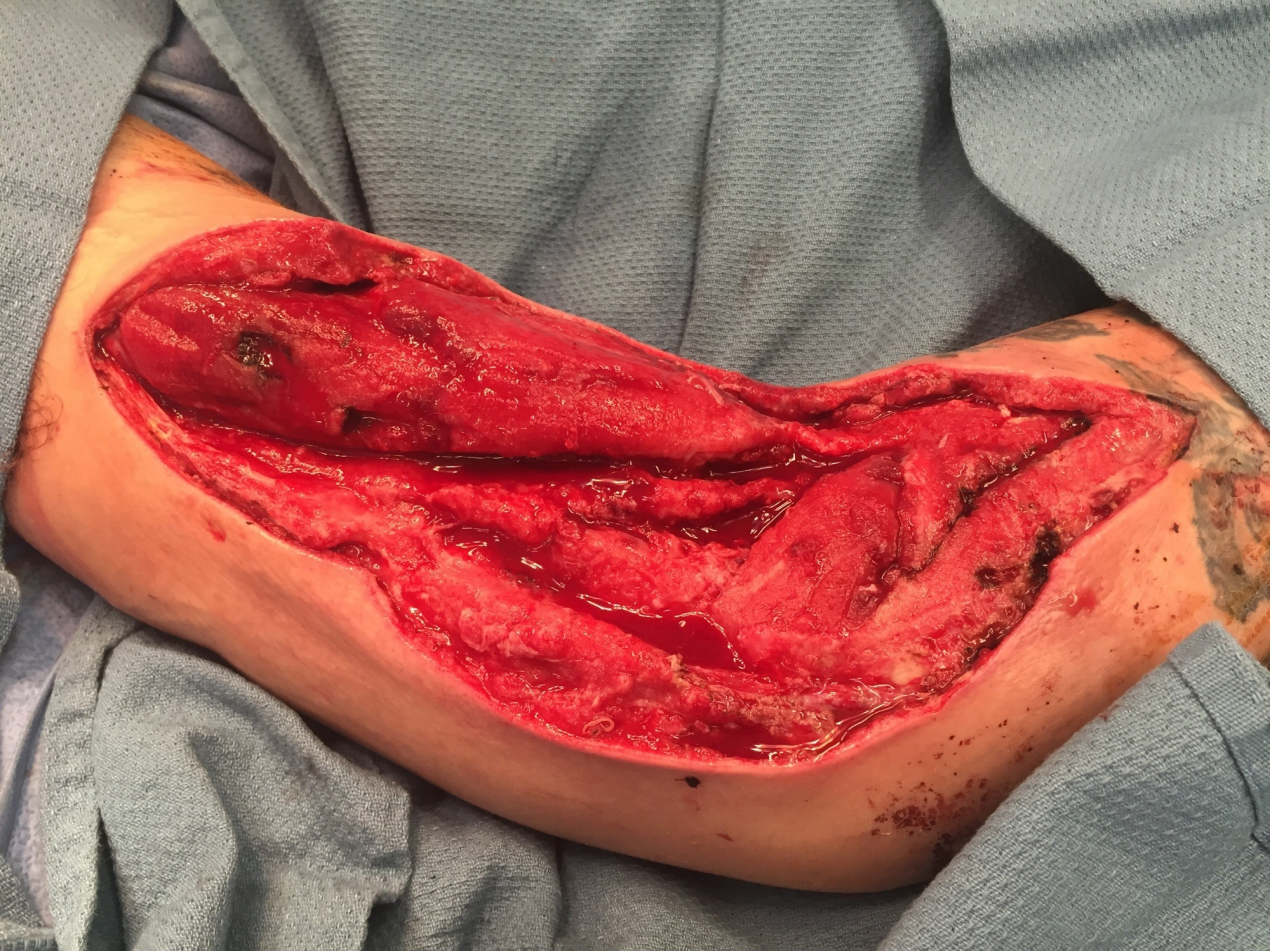
Following NPWTi-d. Note the robust granulation tissue. NPWTi-d: negative pressure wound therapy with instillation and dwell time

**Figure 9:**
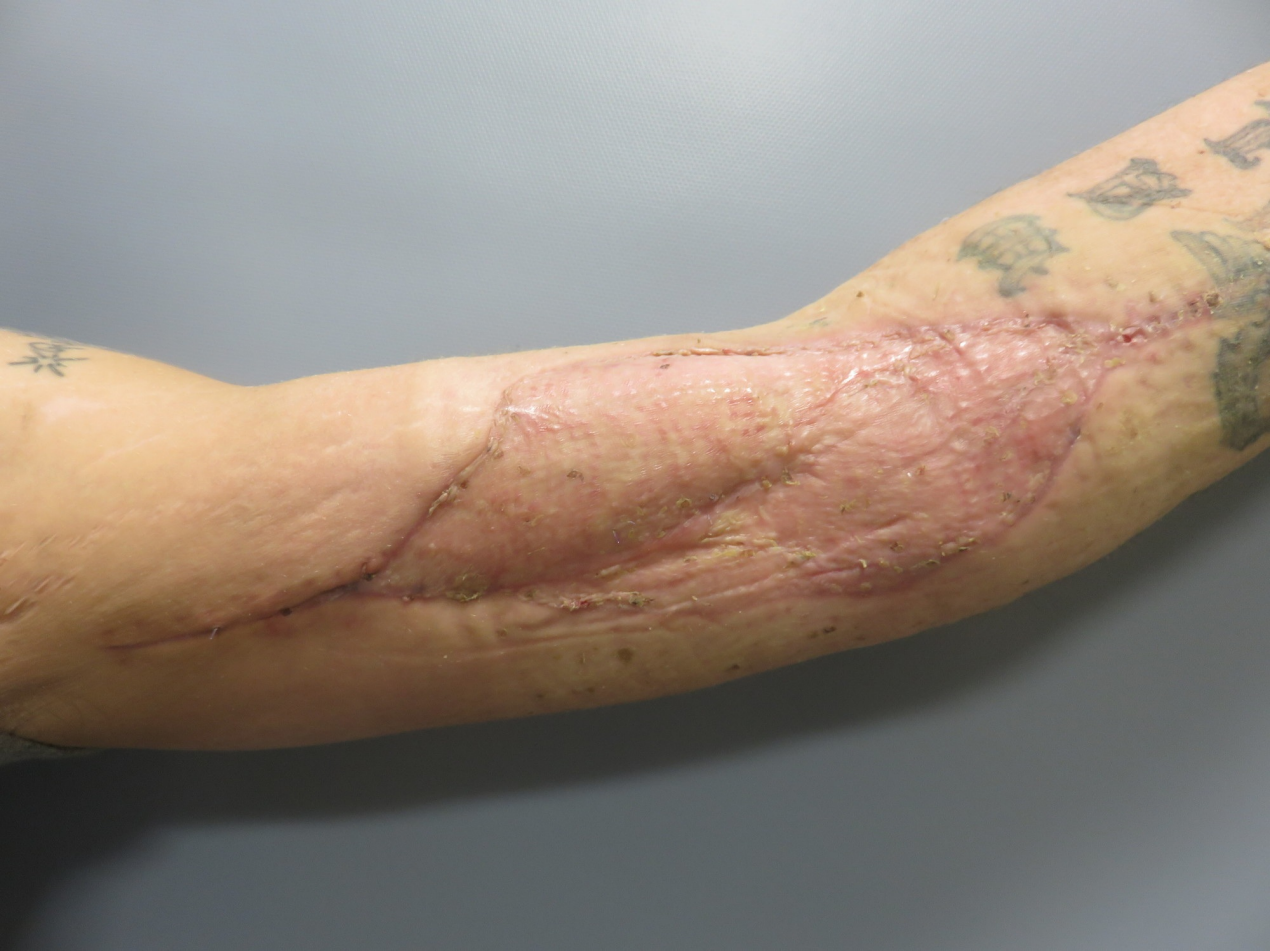
Following muscle transposition and skin graft.

A 31-year-old male with poorly controlled type I diabetes mellitus and a history of intravenous drug abuse presented with a necrotizing soft tissue infection following an upper extremity trauma from a work injury. This was aggressively debrided, leaving him with a grossly swollen and inflamed extremity with a large open wound and exposed brachial vessels. A V.A.C. VERAFLO™ device was placed with a double layer of Adaptic™ non-adhering dressing (Systagenix Wound Management, Acelity, San Antonio, TX ) covering the vessels. This was instilled with 40 cc of normal saline every two hours and allowed to dwell for 10 minutes. Three days later, he presented with massive bleeding for which he was emergently returned to the OR for a saphenous vein patch angioplasty, a biceps brachii muscle flap, a skin graft, and a conventional V.A.C. device. Three days later, he bled a second time requiring an additional saphenous vein patch angioplasty. Six weeks after the injury, his wound had closed and the inflammation had resolved. This case demonstrates how substantial inflammation resolves in association with the use of NPWTi-d (Figures 6-9).

Additional wound hydration

It is widely accepted that moist wounds epithelialize faster than dry wounds. A key property of NPWT is the removal of fluid and optimizing the wound microenvironment through thermal insulation and prevention of evaporative losses [7]. NPWTi-d harnesses the known benefits of wound irrigation and allows for the targeted and repeated even delivery of topical solutions to the wound bed. This additional capacity of NPWTi-d is thought to assist with wound healing.

Case 3: Infected Total Knee

**Figure 10:**
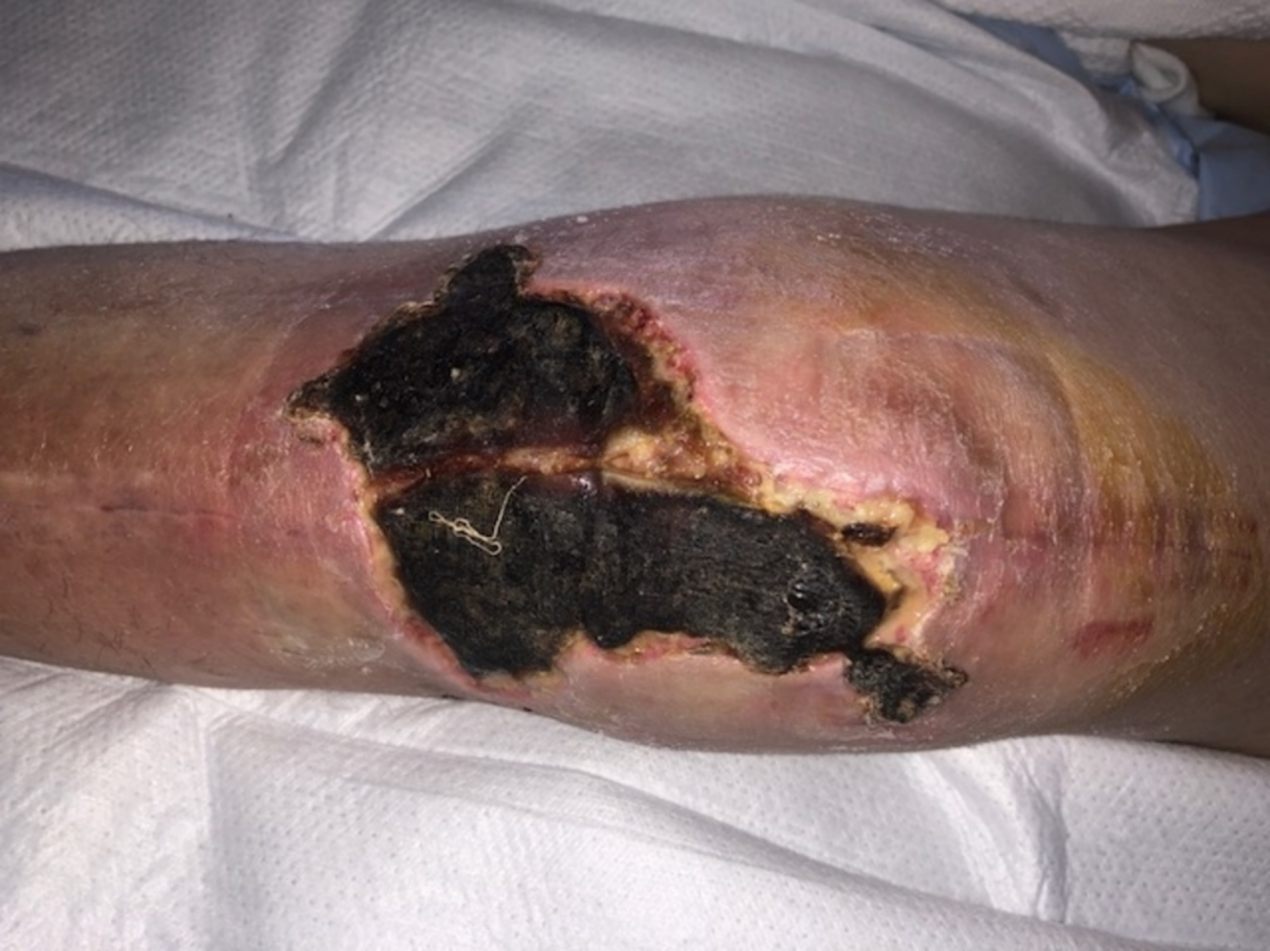
Infected total knee replacement in a patient with diabetes showing a large area of skin necrosis. The patient had undergone multiple previous surgeries on his knee.

**Figure 11:**
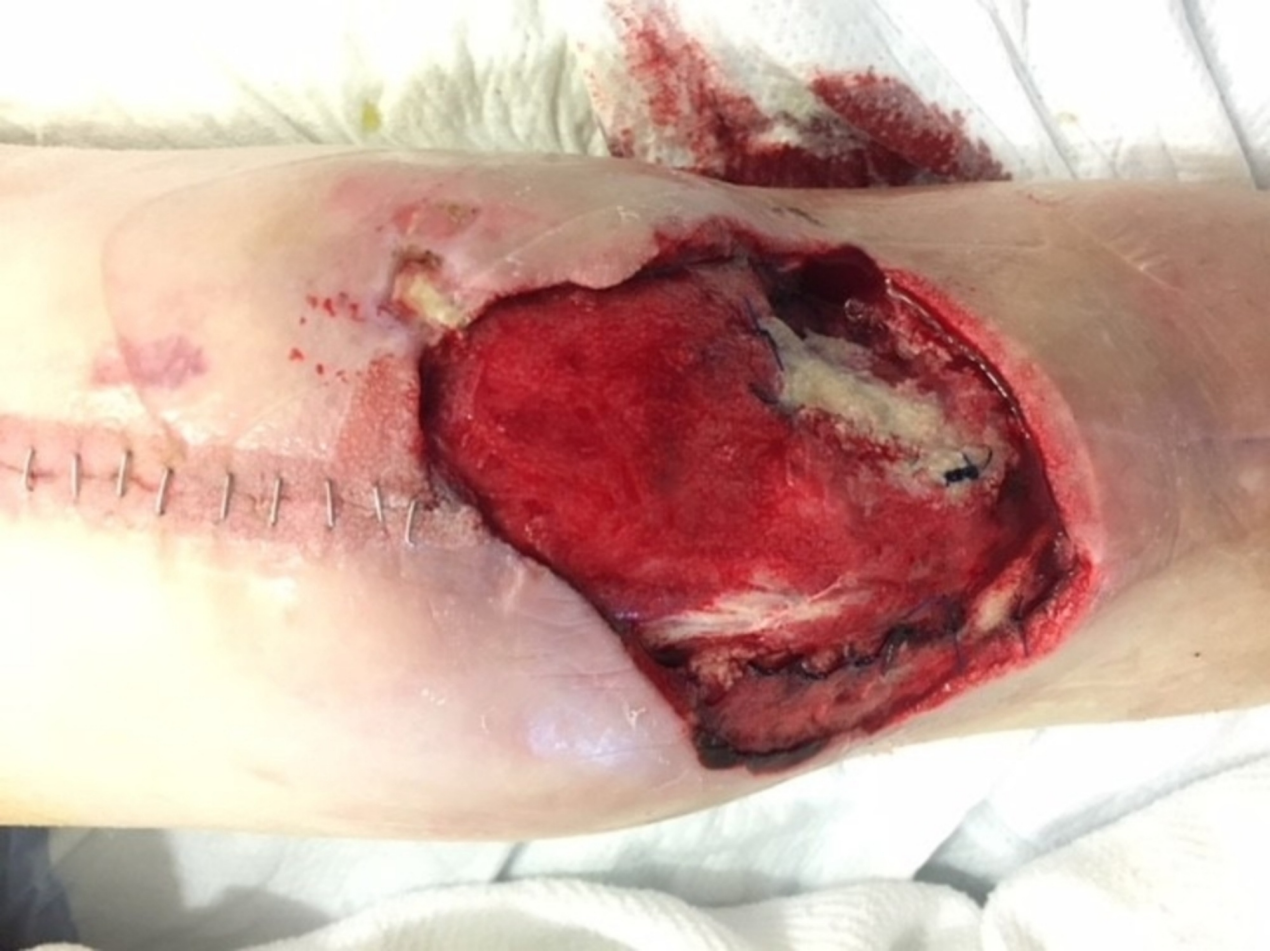
Following excision of necrotic skin and NPWTi-d. Note the rapid development of granulation tissue. NPWTi-d: negative pressure wound therapy with instillation and dwell time

**Figure 12:**
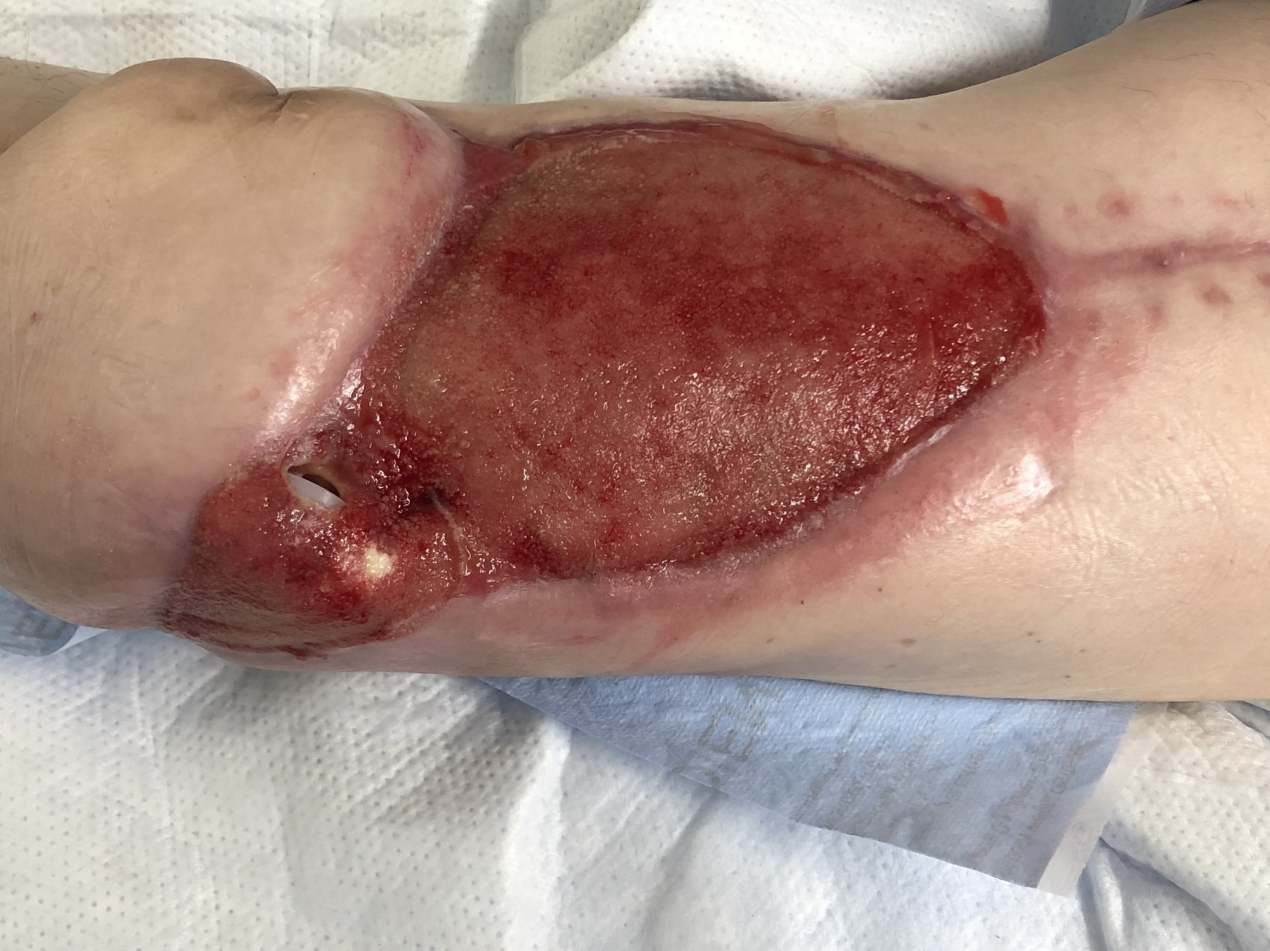
Following perforator flap transfer and treatment of the donor site with Integra Dermal Regeneration Template with V.A.C. Note the new exposure of the patella polyethylene spacer.

**Figure 13:**
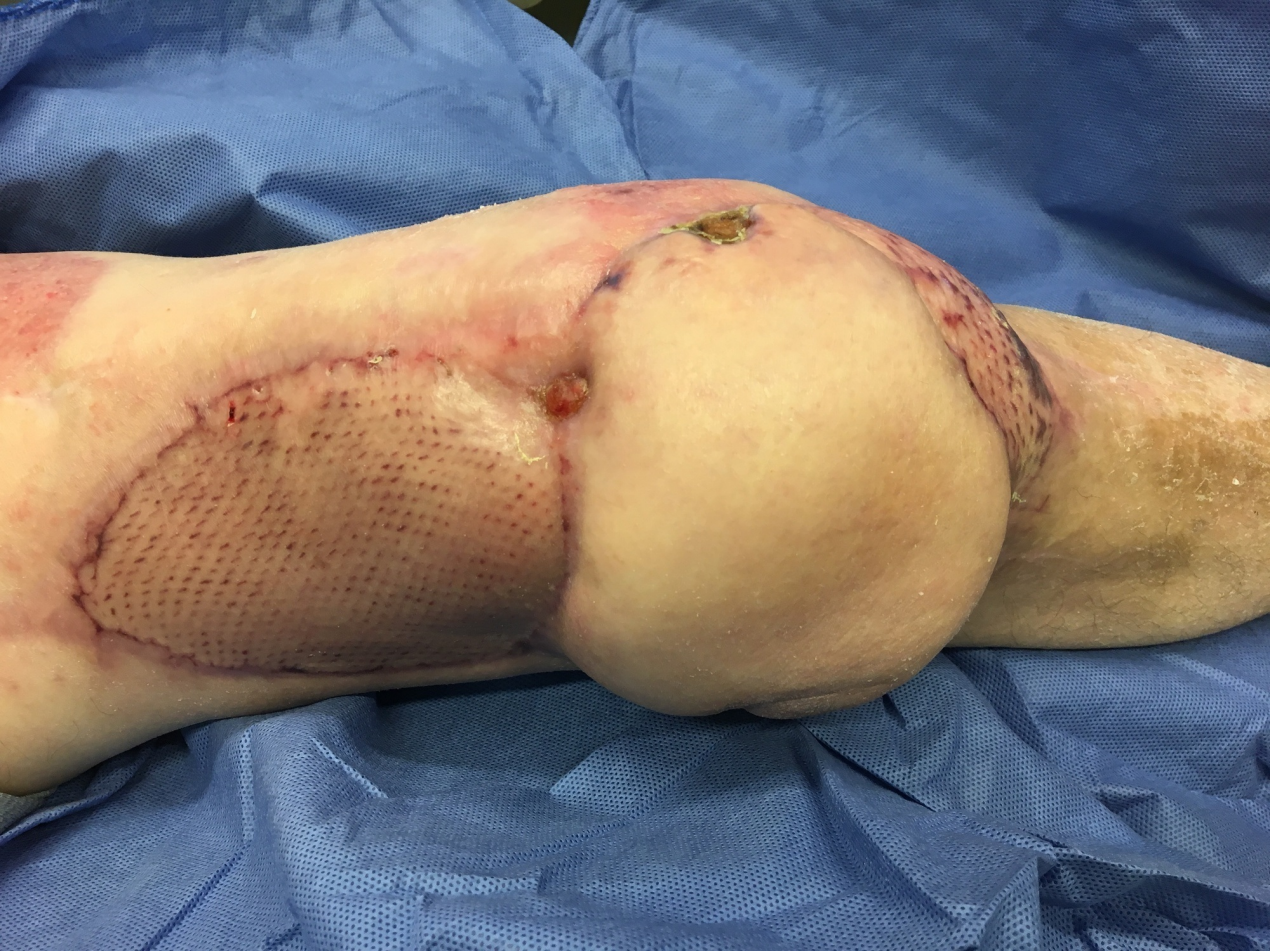
Following additional transposition and skin graft. The polyethylene components had been replaced and washed out.

A 58-year-old male with chronic obstructive pulmonary disease (COPD) and non-insulin-dependent diabetes suffered a fall resulting in a complex left lower extremity compound fracture three years prior. He was treated with an external fixator placement that was complicated by pin site osteomyelitis. Two years later, he developed post-traumatic osteoarthritis of the left knee and underwent left total knee arthroplasty (TKR) and removal of the tibial plate. Approximately three weeks following the TKR, he suffered massive loss of the anterior skin with exposure of the prosthesis. He had multiple previous scars on his legs from his injury and prior surgeries, complicating the flap design, in addition to a tight skin envelope around his knee; his gastrocnemius muscle was also atrophied due to disuse. He was not a free tissue transfer candidate; therefore, we placed him in a V.A.C. VERAFLO™ device and performed a large reverse anterior thigh transposition flap based on a geniculate perforator. This was delayed and transferred in place. The donor site was treated with an Integra® Dermal Regeneration Template (Integra LifeScience Corp., Plainsboro, NJ) with a conventional V.A.C. placement. He developed an additional exposure of his patellar component that required removal, exchange of the polyethylene components, and re-transposition of the flap and skin grafts. Five months following his TKR, he remains in a post-acute care facility. He has a persistent sinus tract below the reconstruction with an otherwise closed wound and continues on suppressive antibiotics. This case illustrates the benefit of maintaining a moist wound, as well as the powerful pro-angiogenic role present with NPWTi-d device (Figures 10-13).

Enhanced angiogenesis and intermittent application

Erba and colleagues showed that NPWT stimulates angiogenesis through several mechanisms, including microdeformation, removal of factors inhibiting angiogenesis, and through the creation of a hypoxia gradient [11]. This gradient in tissue hypoxia upregulates hypoxia-inducible factor-1α and vascular endothelial growth factor (VEGF) expression which ultimately drives microvessel density and directionalized vessel sprouting [11]. Most NPWT systems use continuous suction at 125 mmHg [1-2]. One distinct and potentially important aspect of NPWTi-d is the use of intermittent suction applied after the period of instillation and dwell. In pre-clinical studies, the use of intermittent NPWT was shown to promote more robust granulation tissue and achieve faster wound closure rates [10]. Recent pre-clinical studies investigating these effects in NPWTi-d have also suggested similar findings compared to NPWT continuous and dynamic pressure control (DPC) applications, although robust comparative effectiveness studies are lacking.

Case 4: Radiated Pelvic Exenteration Defect

A 59-year-old female underwent radical vulvectomy and sentinel lymph node biopsy for poorly differentiated squamous cell carcinoma. Her pathology revealed positive margins treated with adjuvant radiation, receiving a total dose of 5,940 cGy. All lymph nodes were negative. One year after completing radiation, she presented with pain along the vulva and gluteal region showing recurrent squamous cell carcinoma.

She underwent a pelvic exenteration, leaving a large surgical wound measuring 30 x 30 cm. Her thigh skin could not mobilize to fill this massive defect, and urinary and colonic diversion precluded the abdomen as a donor site. She was not a free tissue transfer candidate primarily due to her morbid obesity. We, therefore, mobilized the omentum into the pelvis and a large VAC VERAFLO™ device was fit to size. We instilled 100 cc of saline every two hours, allowing it to dwell for 10 minutes. Three days later, the patient returned to the OR where an abdominal advancement flap was created covering 10 cm over the pelvic inlet. The omental flap was found to be viable, and a Polyglactin 910 mesh (Ethicon, Sommerville, NJ) was sutured over the omentum connecting it to the soft tissues over the pelvic opening. The skin in the area was further advanced, substantially reducing the size of her wound. A V.A.C. VERAFLO™ device was again placed. The patient was returned to the OR twice weekly for two weeks for subsequent standard V.A.C. changes and further advancement of local tissues to close her wound. She eventually underwent a large left anterolateral thigh musculocutaneous flap for definitive closure. 

**Figure 14:**
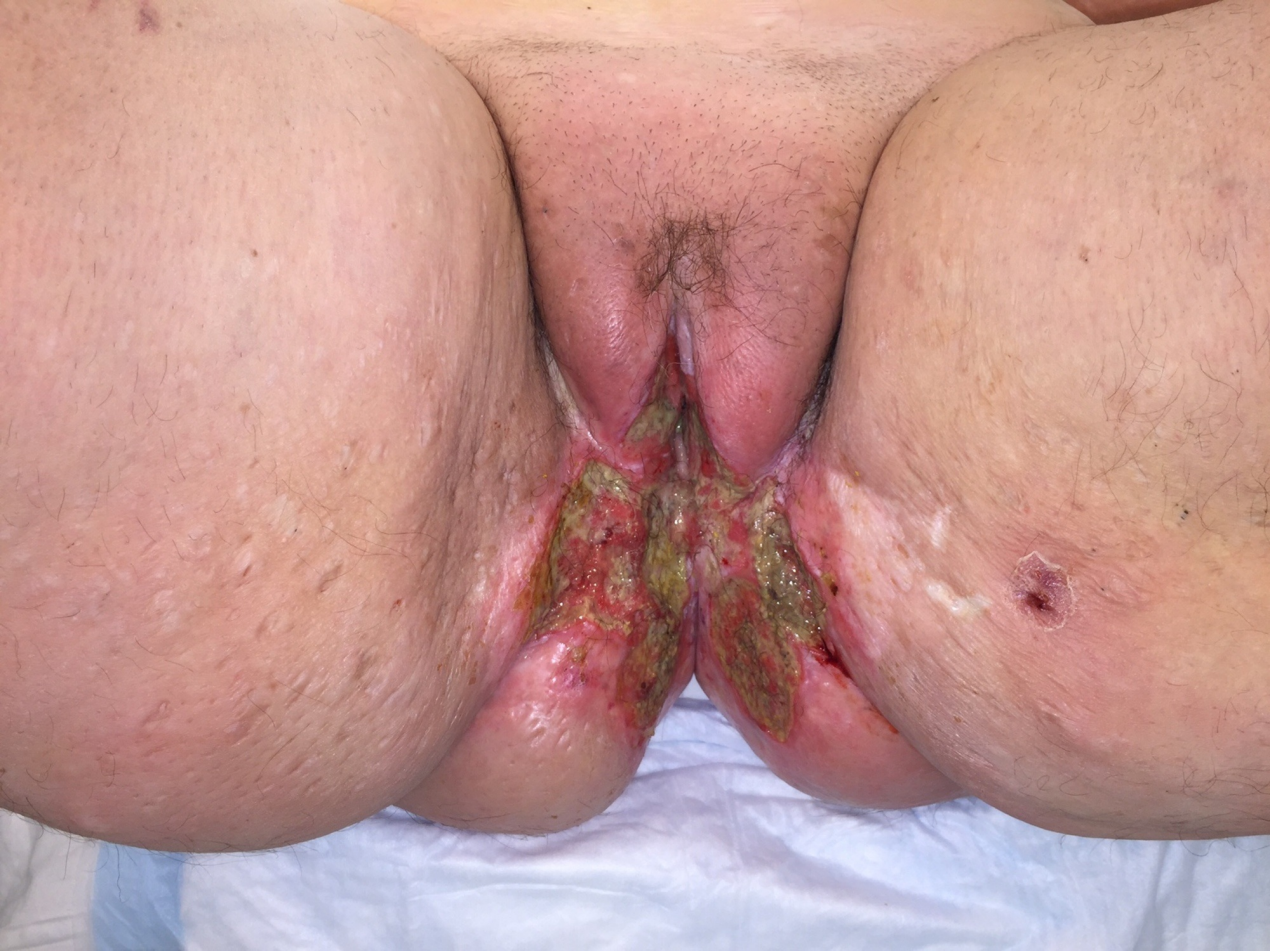
Highly invasive squamous cell carcinoma of the vulva that recurred despite radiation therapy in a morbidly obese female.

**Figure 15:**
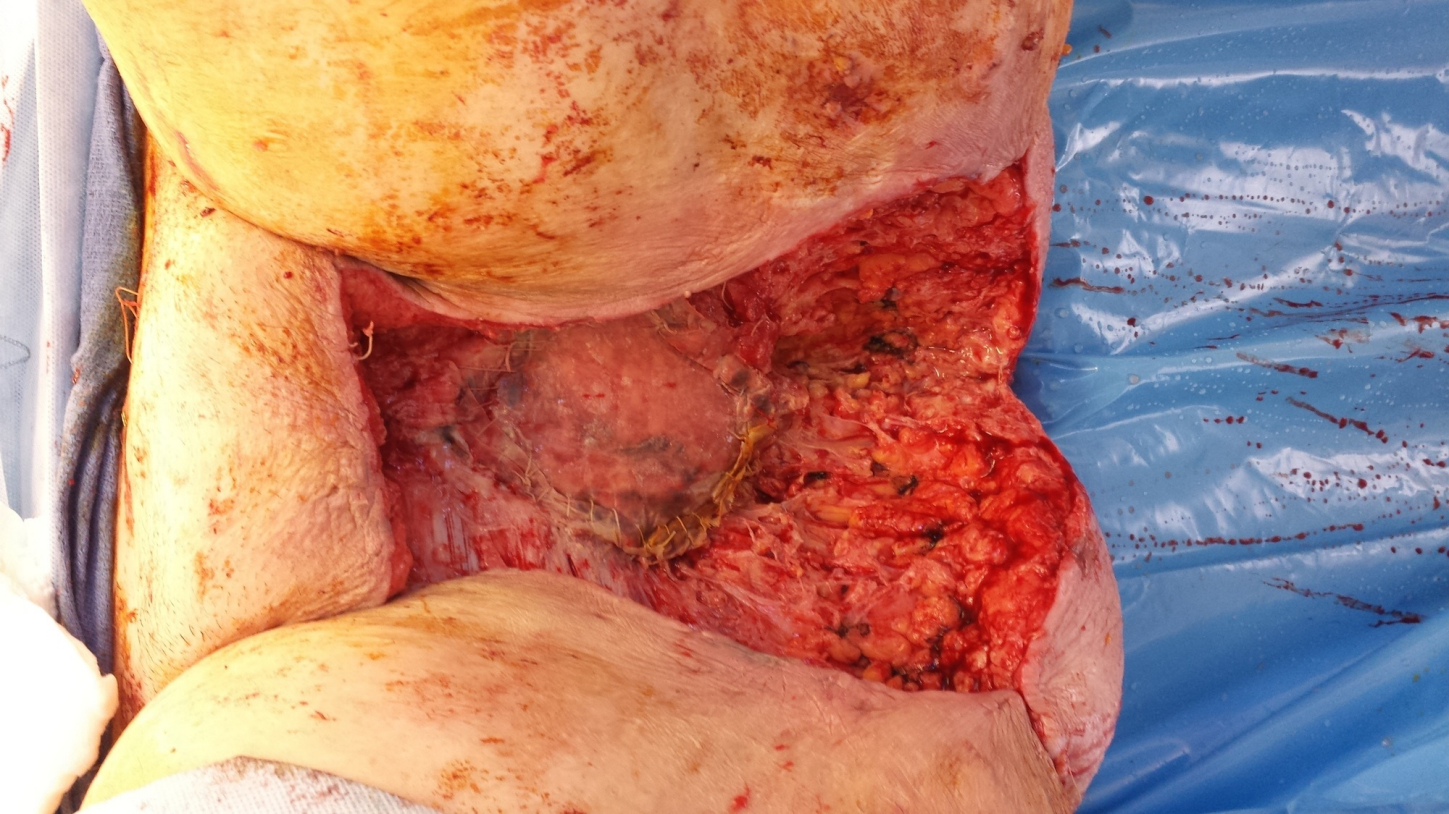
Following surgical resection of the tumor with partial closure. Note the Polyglactin 910 mesh covering an omental flap at the pelvic inlet.

**Figure 16:**
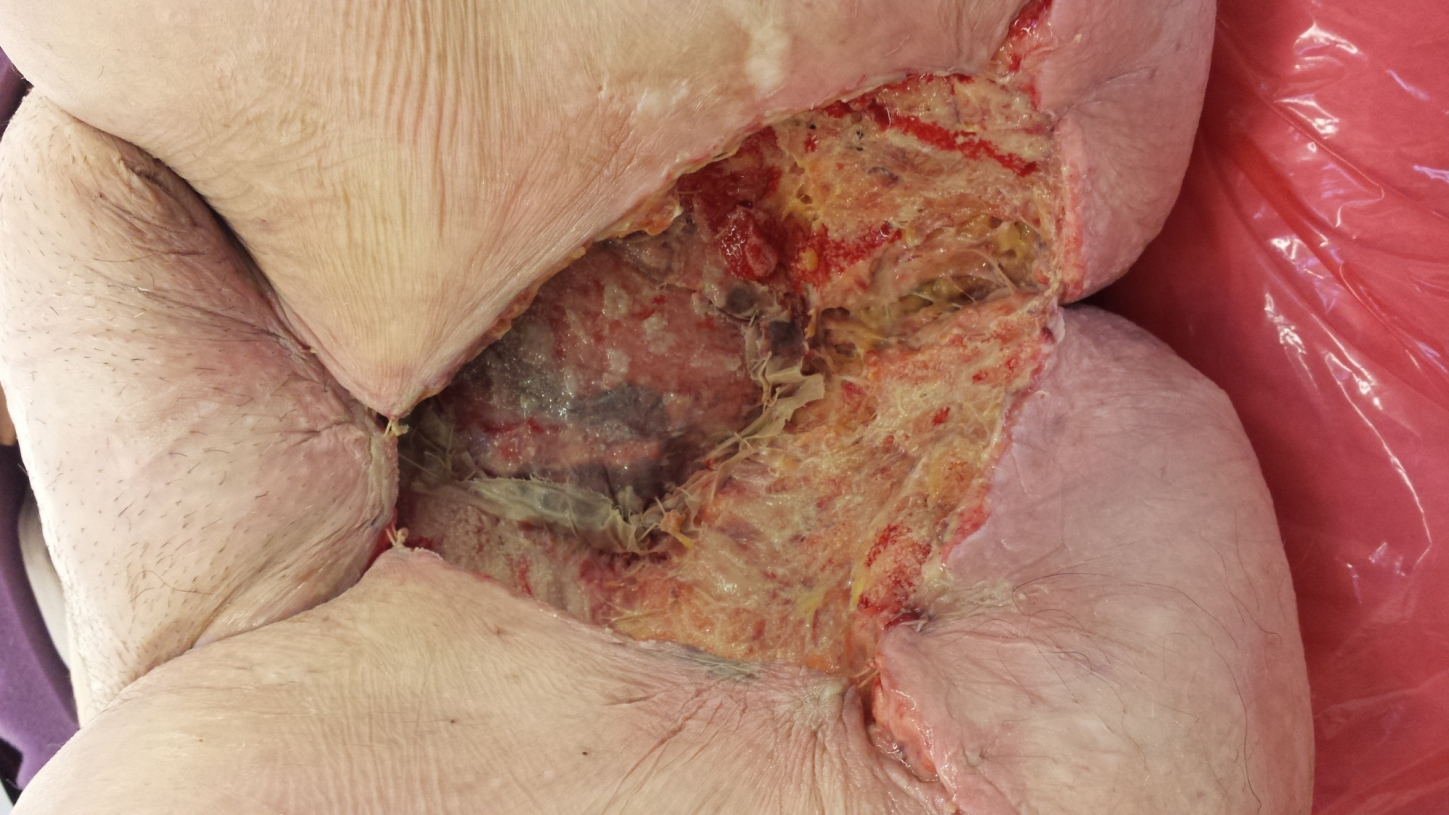
Following treatment with NPWTi-d with a V.A.C. VERAFLO Cleanse Choice Dressing showing honeycomb areas of granulation tissue. Also, note the progressive necrosis of the previously irradiated tissues at the wound edges that required subsequent debridements. This patient had substantial issues with both leakage and pain. NPWTi-d: negative pressure wound therapy with instillation and dwell time

**Figure 17:**
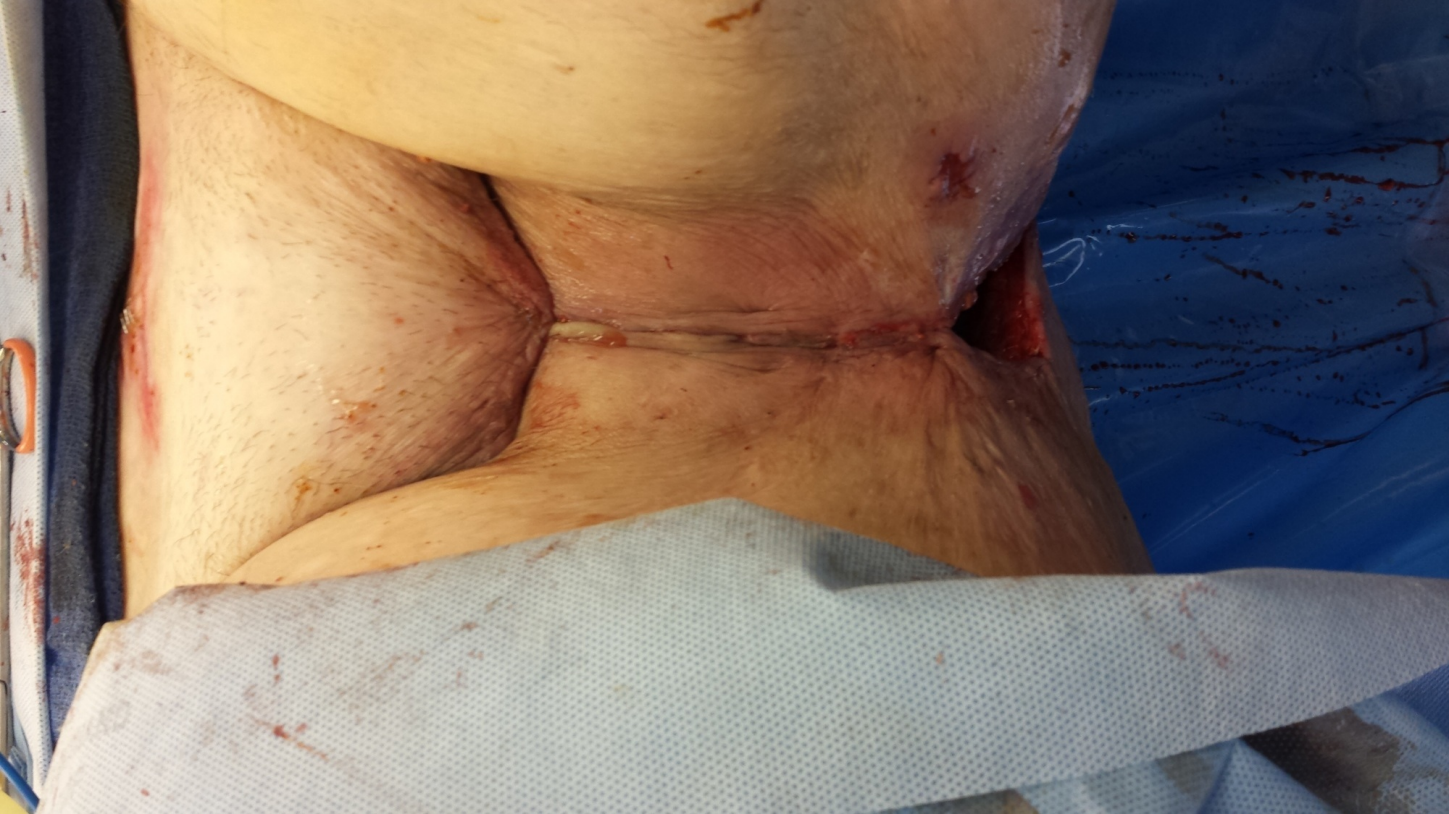
Following surgical closure of the defect.

Unfortunately, approximately six months following pelvic exenteration, she was diagnosed with recurrent inoperable disease. She passed away 11 months following her surgery (Figures 14-17).

Complications encountered

Bleeding

Use of NPWT open reticulated polyurethane foams directly on exposed vessels or at sites at risk of increased bleeding is contraindicated. We followed the recommendation in the literature and our own experience in using a non-adherent layer as a protective barrier over the vessels. As reported in Case 2, despite covering the exposed brachial vessels with two layers of Adaptic™ and placing a lower suction setting of 75 mm Hg, our patient had a major, life-threatening bleeding episode. He did have a second bleeding episode following muscle flap coverage, suggesting that the bleeding was likely due to an inherent infection in the arterial wall.

Maintaining a Seal

Our transition to instillation was notable for several issues, including leakage of the fluid and the ability of the fluid to disperse throughout the foam. The manufacturer (Acelity, San Antonio, TX) has made important changes in product design (V.A.C. Ulta™ System with VERAFLO™), including a skin prep and drape modifications to improve the seal, as well as to create a more hydrophilic foam (polyurethane ester rather than polyurethane ether foam). This foam comes in two varieties with the black foam having a similar pore structure as the classic Granufoam™ foam (Acelity, San Antonio, TX) and a grey foam that is a reticulated open cell foam dressing (ROCF‐CC) (V.A.C. VERAFLO Cleanse Choice™ Dressing) that has a variable pore size and a contact layer with multiple 1.0 cm holes spaced at 0.5 cm in a honeycomb array [14]. These changes have mitigated prior frustrations and shortcomings. Clinicians continue to be challenged by placing NPWT devices in areas of irregular skin contour or near mucous membranes.

Pain

Patients with large complex wounds can have significant pain. Many have some degree of discomfort during the fluid instillation and evacuation phases of this therapy. Some patients have reported difficulty with sleeping. We have discontinued instillation therapy during sleep in some cases.

## Discussion

NPWT has revolutionized the way we care for complex wounds. NPWTi-d is a natural extension of this endeavor and allows for several added benefits using established principles in wound care. While NPWT and NPWTi-d allow us to care for and effectively manage the most complicated cases, these devices are not immune to limitations and failure. In this review, we describe the mechanisms of action, along with our clinical experience which highlights the abilities and drawbacks of NPWTi-d. While more robust pre-clinical and comparative effectiveness studies are needed, NPWTi-d appears to fulfill a unique role and provides an excellent adjunct to the armamentarium in the care of complex wounds.

NPWTi-d has the capacity to clear microorganisms and dilute cytotoxic biomolecules. In addition, by the very nature of instillation and dwell, we know that the suction is temporarily halted during the instillation and dwell phases. This, in essence, causes an intermittent application of suction to the wound. Our previous studies showed that the microdeformation of the wound surface induced by the pores of the foam causes activation of the hypoxia-inducible factor-1α pathway that up-regulates VEGF [11]. We have found that there is a normalized VEGF gradient and that the vessels that form have a more normal morphology compared with more ectatic vessels of an open wound. Presumably, the combination of the intermittent application of suction has a positive effect on the promotion of granulation tissue. 

There is a great need for better higher-level clinical studies, both as registry trials and further prospective randomized clinical trials. Cost-effectiveness analyses will also become increasingly important in our current cost-conscious healthcare environment. Lastly, as more innovative technologies become available, comparative effectiveness studies will be important to try to better understand the optimal use of these devices and to best define their value to patients and the healthcare system.

## Conclusions

NPWTi-d is a technology that adds potential benefits to complex wounds. In our institution, we have used this in challenging wounds and report our early observations that highlight these potential mechanisms of action: 1) facilitated removal of microorganisms, 2) dilution of inflammatory and cytotoxic macromolecules, 3) additional wound hydration, and 4) enhanced angiogenesis through the intermittent application of NPWT. We look forward to further research to help better guide clinicians on the optimal use of this technology in specific disease states.
